# Diet therapy for the treatment of Alzheimer’s disease in view of traditional Persian medicine: A review

**DOI:** 10.22038/ijbms.2019.36505.8694

**Published:** 2019-10

**Authors:** Milad Iranshahy, Behjat Javadi

**Affiliations:** 1 Department of Pharmacognosy, School of Pharmacy, Mashhad University of Medical Sciences, Mashhad, Iran; 2 Biotechnology Research Center, Pharmaceutical Technology Institute, Mashhad University of Medical Sciences, Mashhad, Iran; 3 Department of Traditional Pharmacy, School of Pharmacy, Mashhad University of Medical Sciences, Mashhad, Iran

**Keywords:** Alzheimer’s disease Dementia, Natural products, Saffron, Traditional Persian – Medicine

## Abstract

Alzheimer’s disease (AD) is a neurodegenerative disease and the leading cause of dementia worldwide. Epidemiological studies support the important role of diet in prevention and improvement of AD. In Traditional Persian Medicine (TPM), there is multiple dietary guidelines to prevent and alleviate dementia and memory impairment. Pharmacological studies have been shown that most of the TPM-recommended dietary items can improve memory and cognitive decline and possess anti-amyloidogenic, *etc*. activities. Among them, garlic (*Allium sativum*) and its compounds, S-allyl-cysteine and diallyl-disulfide, coconut (*Cocos nucifera*) oil, saffron (*Crocus sativus*) and crocin and crocetin, honey, fish, lemon balm (*Melissa officinalis*) and its major compounds rosmarinic acid, raisin and resveratrol, rose flowers (*Rosa damascna*) and geraniol, ginger (*Zingiber officinale*) and its 6-gingerol and 6-shogaol, cumin (*Cuminum cyminum*) and its main component cuminaldehyde have been found to possess stronger anti-AD activities. Most of these items exhibited antioxidant and AChE inhibitory activities and decreased lipid peroxidation. They also possessed anti-amyloidogenic effects, reduced cerebral plaques and Aβ-species, suppressed cerebral inflammation and alterations in tau protein and inhibited Aβ-induced apoptosis through various mechanisms. Noticeably, there are similarities between TPM anti-AD diet and the typical Mediterranean diet whose beneficial effects on AD have been widely demonstrated. Given the importance of traditional medicine systems in discovering new medicines and nutraceuticals for curing ailments, considering TPM anti-AD dietary recommendations in future research would be helpful.

## Introduction

Alzheimer’s disease (AD) is a progressive neurodegenerative disease and the leading cause of dementia. The most common symptom of AD is early memory decline and cognitive deficit. This memory loss is caused by malfunction and death of brain neurons involved in forming new memories (1-3) .

Currently, over 36.5 million people are affected by dementia worldwide, the majority of which are AD-related. Approximately, 5–7 million new AD cases are being diagnosed yearly in the elderly population (4). It is estimated that the worldwide prevalence of AD will triple by 2050 (5). More importantly, the high economic burden of AD would be affecting patients and their family and community. 

AD develops as a result of multiple factors including aging over 65 and having apolipoprotein E (APOE) ε4 gene, family history, mild cognitive impairment (MCI), cardiovascular disease risk factors (e.g. smoking, obesity in midlife and diabetes, midlife hypertension) and lower educational attainment (1).

The core cerebrospinal fluid (CSF) biomarkers of AD are remarkable increase in T-tau and P-tau along with a considerable decrease in Aβ42 (6). Moreover, the presence of senile plaques comprised of amyloid-β (Aβ) peptides and neurofibrillary tangles (NFTs) comprised of hyperphosphorylated tau are other important features of AD (7, 8). 

In view of the high prevalence AD and the increasing burden of dementia and the lack of progress in developing an effective medication for AD, searching for new drugs seems to be of ample significance. 

Epidemiological studies point out the important role of diet in prevention and improvement of AD (9). In Traditional Persian Medicine (TPM), there exist multiple dietary guidelines to prevent and alleviate dementia and memory impairment. Eminent Persian physicians including Ibn-Sina (Avicenna; 980–1037 AD), Razi (Rhazes; 854–925 AD) Jorjani (1042–1136 AD), and Aqili Khorasani (18^th^ century) have discussed dementia and its etiology along with medicinal and nutritional strategies for treatment of the disease in their medical textbooks (10-12). Given the great importance of various traditional medical systems in identifying new medications and nutraceuticals especially in recent decades, considering TPM-reported food items would be useful in finding new drugs and dietary sources for the treatment of different ailments. 

Herein, we aim to study TPM-recommended plant and animal-based food items for the treatment of dementia. In addition, epidemiological and pharmacological evidence supporting the effects of TPM-recommended foods in preventing and improving cognitive and memory performance will be discussed.

## Methods

TPM anti-dementia food items were obtained from major TPM texts including Canon of Medicine by Avicenna (12), Al-Hawi fi’l-Tibb (The Continence) by Rhazes (10), Kamel al-Sina’ah al-Tibbiyah (The Perfect Art of the Medicine) by Ahvazi (13), Zakhireh Kharazmshahi (Treasure of Kharazmshahi) and Al-Aghraz al-Tibbiah (Medical Goals and Allaii’s Discussions) by Jorjani (11), Hedayat al-Mota’allemin fi al-Tibb (An Educational Guide for Medical Students) by Akhawayni (14), Makhzan al-Adwiah (Drug Treasure) by Aqili (15) and Exir-e-Azam by Chashti (16). Following Persian and Arabic keywords were used to search the mentioned TPM textbooks:“Nesyan”, “Faramoshti”, “Faramoshtkari”, “Taqviat hafezeh”, “Fesaad al-zekr”, “Fesad al-hefz” and “Alhefz”.

The equivalent scientific names of the obtained foods were authenticated using botanical encyclopedias, including the ‘Illustrated polyglottic dictionary of plant names in Latin, Arabic, Armenian, English, French, German, Italian, and Turkish languages’ by A. K. Bedevian (17), ‘Dictionary of Medicinal Plants’ by A. Soltani (18), ‘Explanation of Dioscorides’ Book’ by Ibn-Beythar, ‘Qamus al-qanun fi’l tibb’ by Abdol-hamid, and ‘Encyclopedia of Medicinal Plants: Arabic–English–French–German–Latin’ by M. Hayek (19). In the next step, an extensive search on scientific databases such as Google scholar, Pubmed, Scopus and Science direct was carried out using the TPM-reported foods of herbal and animal source to retrieve epidemiological and pharmacological studies supporting their anti-AD properties together with the relevant mechanisms of action. 

**The pathophysiology of AD**

Fibrillar Aβ is the main component of amyloid plaques in the brains of AD patients (20). Two common isoforms of Aβ are Aβ40 (the more common form) and Aβ42 (the more amyloidogenic but the less common form) (21).

A protease enzyme is α-secretase that cleave amyloid precursor protein (APP) to produces the non-amyloidogenic soluble amyloid precursor protein α (sAPPα) (22). sAPPα have been shown to promote the growth of cultured neurons under physiological and non-physiological conditions such as Aβ toxicity and glucose or oxygen deprivation (23). In AD, during amyloidogenic process, Aβ is generated through the cleavage of APP by β- and γ-secretases (22). In this pathway, β-secretase also known as β-site amyloid precursor protein cleaving enzyme 1 (BACE1), first cleaves the transmembrane APP protein to generate sAPPβ (an extracellular fragment) and APP-β-CTF (C99; a membrane-associated carboxyl-terminal fragment). APP-β-CTF is then cleaved by γ-secretase to form Aβ fragments which are then released into the extracellular space (24). Consequently, Aβ peptides aggregate into soluble oligomers and then unify to produce insoluble β-sheet fibrils which are deposited in diffuse senile plaques (25).

Given the important role of β-and γ-secretases in Aβ generation, inhibition of these enzymes would be a principle therapeutic target for decreasing cerebral Aβ levels in patients with AD. Activation of α-secretase is also considered a therapeutic strategy for Aβ reduction (26). 

Tau proteins are proteins that serve to stabilize microtubules. However, tauopathy plays important role in AD pathogenesis (27). Alterations in the brain levels of tau or its structure can affect its physiological function. Glycogen synthase kinase 3 (GSK3)-mediated hyperphosphorylation of tau could lead to self-aggregation into paired helical filaments (PHFs). These tau assemblies may result in loss of neuronal synaptic contacts followed by neuronal death and lysis which in turn causes the release of tau into the extracellular space. The liberated tau then binds to molecules like sulfoglycosaminoglycans (sGAG) which promote its polymerization. Upon glycation, tau polymers could aggregate to form NFTs which ultimately lead to neuron loss in AD (28). 

Accumulating evidence has demonstrated that dysregulated neuronal cyclooxygenase-2 enzyme (COX-2) activity may affect normal neuronal activity, resulting in cognitive impairment. In fact, PGE 2 and soluble Aβ 42 oligomers act in synergy to disrupt hippocampal long-term potentiation (LTP)(29). PGE 2 also mediates the potentiation of γ-secretase activity and subsequently, Aβ peptide generation (22). Moreover, memory deficits has been shown to be aggravated by increased COX-2 activity in APP-PS1 mice ((29). Therefore, COX inhibitors can be beneficial in the management of AD.

Acetylcholinesterase is a hydrolase enzyme that hydrolyzes choline esters such as acetylcholin and possesses a crucial role in acetylcholine-mediated neurotransmission (30). It is evidenced that plasma AChE activity is increased in subjects with early AD. Moreover, AChE may possibly play a role in Aβ fibrillogenesis (31). 

Microglia, the brain’s tissue macrophage, are key players of the immune and inflammatory responses of the CNS. Microglia have the responsibility to provide immune surveillance and migrate in response to pathogens and injuries (32). Moreover, under pathological conditions, the activation of microglia maintains CNS homeostasis. However, during aging, chronic microglial activation could damage neurons through the release of proinflammatory cytokines. Thus, suppressing microglial activation is considered a potential therapeutic goal in AD management (33).

Currently, only a few drugs including AChEIs, rivastigmine, galantamine, tacrine and donepezil and a NMDA receptor antagonist, memantine are approved by US Food and Drug Administration (FDA), for the treatment of cognitive symptoms of AD (25).

**Plant- and animal-based foods recommended by TPM for treatment of dementia and memory impairment**

Several plant foods and animal products have been suggested by TPM to be effective in prevention and treatment of memory loss. Plant-based TPM food items are listed in Table 1. TPM-reported animal-based food items to cure memory impairment include whey, fish, fish eggs, egg yolk, birds meat and brain, lamb and kid (goat) meat, cow and gout milk, barley soup, and honey (10-12, 16, 34).

***Allium sativum*** **L.**

Garlic, a member of Amaryllidaceae, has long been used as a food seasoning and a medicinal plant (35). Several studies reported neuroprotective and anti-inflammatory activities of garlic (32). *In vitro*, ethyl acetate fraction of aged garlic extract exhibited a high 2,2’-azino-bis (3-ethylbenzthiazoline-6-sulfonic acid) diammonium salt (ABTS) radical scavenging and malondialdehyde (MDA) inhibitory activities. Moreover, ethyl acetate fraction significantly reduced Intracellular reactive oxygen species (ROS) accumulation induced by Aβ treatment in PC12 cells. The fraction also protected PC12 cells against Aβ-induced neurotoxicity (35).

**Figure 1:**
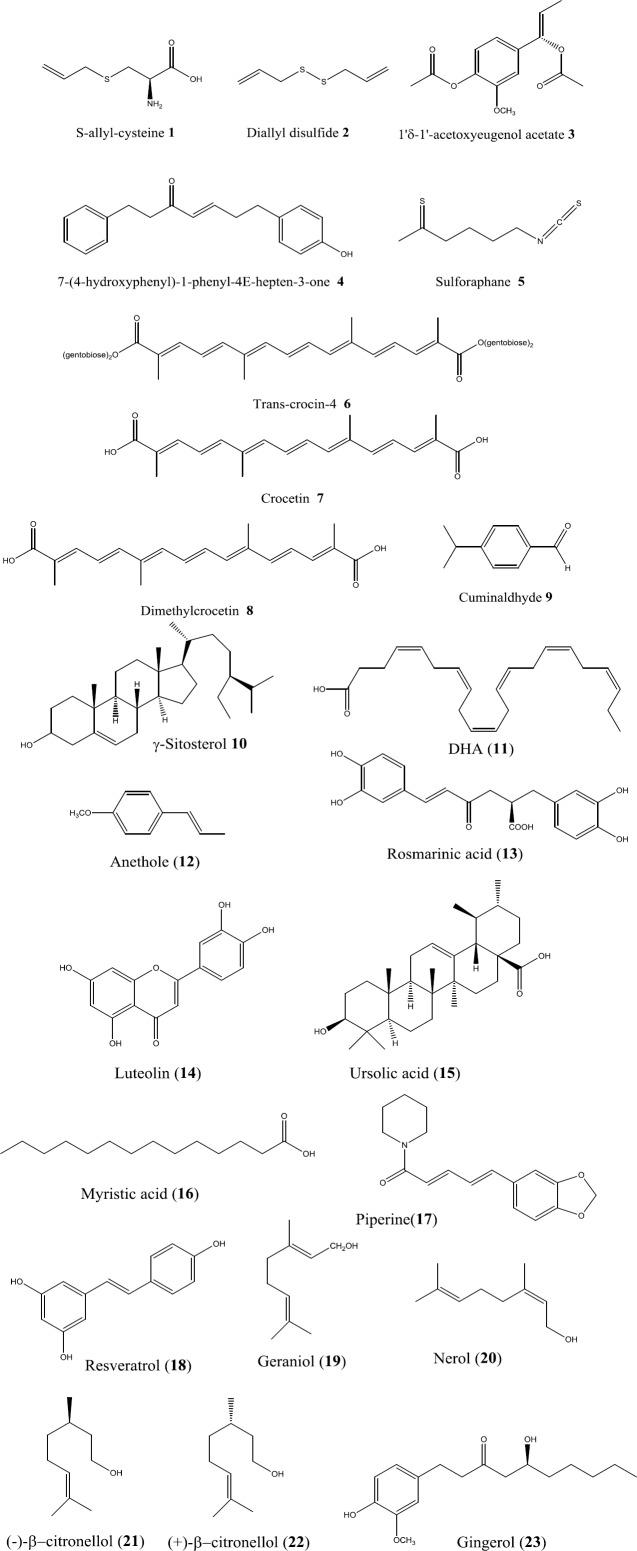
Chemical structures of natural compounds with anti-Alzheimer's disease activity

Chauhan *et al*. investigated the anti-amyloidogenic effects of dietary supplementation with aged garlic extract (40 mg/kg/d for 4 weeks) in a mouse model of AD that overexpresses the human amyloid precursor protein 695 carrying Swedish double mutation (K670N/M671L) (Tg2576). Results show 64% reduction in cerebral levels of sAPPa, and ∼21-fold elevation of Aβ40 and Aβ42 in untreated Tgs compared to wild type and littermate controls. Dietary garlic resulted in a 25% increase in sAPPa levels and 31% and 32% decreased in Aβ40 and Aβ42, compared to untreated Tgs (36). In another experiment, dietary aged garlic extract (2%) and its main compounds S-allyl-cysteine (Figure 1, **1**) and diallyl-disulfide (**2**) (20 mg/kg) reduced cerebral plaques, soluble and fibrillar Aβ-species, cerebral inflammation, and phosphorylation-induced conformational alteration in tau protein (via GSK-3β inhibition) (37). Moreover, the aqueous extract of fresh garlic not only inhibited the formation of Aβ fibrils in a concentration and time dependent manner but was also cable of defibrillating Aβ fibrils. The boiled aqueous garlic extract also retained its anti-amyloidogenic activity while its fibril degrading activity was significantly lost which suggests that anti-amyloidogenic activity of garlic is non-enzymatic (38). 

Jeong *et al* investigated the effects of ethyl acetate fraction from aged garlic ethanol extract (5, 10 and 20 mg/kg) on Aβ-induced memory and learning impairment in mice using Y-maze test and passive avoidance task. It was shown that ethyl acetate fraction attenuated memory impairment in a dose-dependent manner. It also improved the Aβ-induced deficit in the passive avoidance test (35).

***Alpinia officinarum*** **Hance** ***, Alpinia galanga (*** **L.** ***)*** **Willd**

Rhizomes Galangals (Zingiberaceae) are plants traditionally used in cuisines and as a medicine for a variety of diseases. 

Singh *et al*. investigated the effects of different fractions of *A. galanga* rhizomes (n-hexane, chloroform and ethyl acetate at 200 and 400 mg/kg) on cognitive performance of amnestic mice (Aβ_(25–35)_-induced AD type) using open field and water maze tests. All fractions could Increase habituation memory and decrease escape latency which indicates the cognitive enhancement. AChE level in brain tissue homogenate was also decreased after treatment with *A. galanga*. Increased levels of Na+/K+-ATPase and antioxidant activity suggests improvement in brain membrane integrity and free radical scavenging activity. Chloroform fraction which contains mostly 1′δ-1′-acetoxyeugenol acetate (**3**), was found to be the most potent fraction (39). Total extract of *A. officinarum* also exhibited 41.3% AChE inhibition at concentration of 300 µg/ml (40).

7-(4-hydroxyphenyl)-1-phenyl-4E-hepten-3-one (AO-1) (**4**), a diarylheptanoid isolated from *A. officinarum*, has been found to possess strong effects on neuronal differentiation and neurite outgrowth. Moreover, AO-1 was able to reduce apoptotic levels and oxidative stress induced by Aβ. The compound also exhibited anti-caspase and dendrite protective activities via activation of phosphatidylinositol 3-kinase (PI3K)-mammalian target of rapamycin pathways (41). 

***Brassica nigra L.***

Black mustard seeds are commonly used as a spice worldwide. It is also used for the treatment of a number of CNS diseases (42). Jazayeri *et al*. reported that seeds of mustard exhibited moderate AChE inhibitory activity with an IC_50_ value of 135.0 ± 5.91 μg/ml (43).

Sulforaphane (**5**) is an isothiocyanate present in mustard. Kim *et al*. showed that sulforaphane was able to ameliorate cognitive function in Aβ-induced acute AD mouse models of Y-maze and passive avoidance behavior. However, this effect was not associated to inhibition of Aβ aggregation (44). 

***Cicer arietinum*** **L.**

Chickpea is an important food legume from Fabaceae which is considered as a good source of dietary protein (45). In TPM, chickpea has been used as a valuable functional food and a medicinal plant.

Wahby *et al*. investigated the protective effects of the isoflavones fraction obtained from chickpea extract (10 mg/Kg for 6 weeks) against AlCl_3_-induced neurodegeneration. The results showed that chickpea attenuated the expression of inflammatory cytnokines, suppressed amyloidogenesis, and maintained the AChE activity and ER-β expression. It also attenuated the oxidative stress and ameliorated the histological changes induced by AlCl_3_ (46).

It has been found that chickpea is a rich source of folate. Several studies revealed that low plasma levels of folate were associated with AD (47). It has been suggested that folic acid deficiency could impair neural DNA repair, and subsequently sensitize the neurons to oxidative damage induced by Aβ (48).

***Cinnamomum cassia*** **(L.) J.Presl**

Aqueous cinnamon extract has been found to inhibit the formation of Aβ oligomers and prevent the toxicity of Aβ on neuronal PC12 cells. In an AD fly model using *Drosophila melanogaster*, the extract improved the reduced longevity, fully recovered impaired locomotion and destroyed tetrameric species of Aβ in brain of the flies. Moreover, cinnamon extract (100 µg/ml of the extract in drinking water) markedly decreased 56 kDa Aβ oligomers, reduced plaques and improved cognitive behavior in a transgenic aggressive mice model (49).

It is stablished that insulin resistance causes memory impairment. Anderson *et al*. investigated the effects of cinnamon-enriched diet on behavior, insulin signaling and AD-associated mRNA expression in the brain of rats fed a high fat/high fructose diet (HF/HFr) to induce insulin resistance for 12 weeks. The results showed that decreased insulin sensitivity induced by HF/HFr was reversed in cinnamon-fed animals. Moreover, cinnamon-fed rats showed more activity in a Y maze test than control and HF/HFr diets-fed animals. HF/HFr diet caused more anxiety in elevated plus maze task which was alleviated by cinnamon. HF/HFr diet also resulted in down-regulation of the mRNA encoding glucose transporter 1 (GLUT1) and GLUT3, and an increase in AD-associated mRNA including phosphatase and tensin homolog (PTEN), tau and APP in the hippocampus and cortex which were alleviated by cinnamon. Peripheral insulin sensitivity was also increased by cinnamon treatment. These finding suggest that cinnamon could improve body insulin sensitivity and related alteration in the brain (50).

***Cocos nucifera*** **L.**

Coconut, the drupe of *C. nucifera* (Arecaceae), has a long history of use as a food, cosmetic agent and medicine. In TPM, It has been considered a medicinal food for preventing and curing many CNS illnesses (10).

Brains of ovariectomised rats can display features similar to those observed in menopausal women with AD. Radenahmad *et al*. investigated the effects of young coconut juice on the pathological alterations occur in the brains of AD ovariectomised rats. Four groups of rats included sham-operated, ovariectomised, ovariectomised +oestradiol benzoate and ovariectomised + coconut juice. Brain sections were immunostained with Aβ_1–42_, glial fibrillary acidic protein (GFAP) (an intermediate neurofilament of astrocytes) and Tau-1 antibodies which are reliable biomarkers of amyloidosis, astrogliosis and tauopathy. The results showed that coconut treatment restored the serum oestradiol to levels significantly higher than that of the ovariectomised and sham groups. Aβ deposition and GFAP expression was significantly reduced in the cerebral cortex of the coconut-treated animals, as compared with the other groups. Tau-1 expression was also suppressed in the hippocampus (51).

Coconut oil which is extracted by either hot or cold pressed techniques, contains tocotrienols, squalene, tocopherols and sterols. Coconut oil consists 92 % saturated fatty acid, with 62–70 % being medium-chain triglyceride which can be rapidly metabolized to form ketones or ketone bodies. Ketogenic diets have been found to be effective as a therapy for neurodegeneration (52).

In a clinical trial, De *et al*. investigated the cognitive impact (orientation, language-building, fixing, calculation-concentration and memory areas) of coconut oil in AD patients through cognitive test Mini-Mental State Examination. For 21 days, half of 44 patients with AD received 40 ml coconut oil daily divided between breakfast (20 ml) and food (20 ml). Cognitive performance in patients who received coconut oil, significantly improved in the orientation and language-construction areas (53). 

***Corylus avellana*** **L.**

*C. avellana* (Betulaceae) edible nuts commonly known as hazelnut has long been considered a “brain-food”. It is highly prescribed as a neuroprotective and to prevent brain atrophy and memory loss in TPM. 

In an experiment, the effect of hazelnut diet [(without skin) 800 mg/kg/day for 1 week] on memory (using Y-maze test and shuttle box apparatus), anxiety (using elevated plus maze task), neuroinflammation and apoptosis was evaluated in Aβ-injected rats. The results showed that hazelnut supplementation improved memory, and reduced anxiety-related behavior. Moreover, Western blot analysis of COX-2, IL-1β, TNF-α, B-cell lymphoma 2 (Bcl-2), Bcl-2-associated X protein, and caspase-3 showed that hazelnut can ameliorate Aβ-induced neuroinflammation and apoptosis (54).

***Crocus sativus*** **L.**

Saffron is the stigma from a bulbous plant from Iridaceae which has been widely used as a food spice, coloring and flavoring agent, and medicinal plant in many cultures., saffron consumption as a food and beverage add (55) In TPM, saffron was traditionally used to alleviate several CNS and mental diseases such as AD, depression, anxiety, tension and insomnia and also as a cardioprotective medicine(56, 57).

Papandreou *et al*. investigated the effects of an aqueous: methanol (50:50, v/v) extract of saffron stigmas on Aβ-aggregation and fibrillogenesis using thioflavine T-based fluorescence assay and by DNA binding shift assay. The results showed that saffron inhibited Aβ fibrillogenesis in a concentration and time-dependent manner. Trans-crocin-4 (**6**), the digentibiosyl ester of crocetin (**7**) and the main carotenoid present in saffron, inhibited Aβ fibrillogenesis at lower concentrations than dimethylcrocetin (**8**), indicating that glycosylated carotenoid is more active (58). The results of 8-anilinonaphthalene-1-sulfonic acid (ANS)-binding assay showed that crocin, a carotenoid responsible for the color of saffron (59), decreased the hydrophobic area in incubated Aβ42 which is accompanied by a structural change to α-helical and β-turn. This indicates that the anti-amyloidogenic effect of crocin might be due to the inhibition of Aβ formation along with breaking down amyloid aggregates (21). Crocin was capable of decreasing Bax/Bcl-2 ratio and cleaved caspase-3 level which indicates that crocin inhibits Aβ induced apoptosis possibly by its antioxidant activities (60).

Khalili *et al*. investigated the effect of crocin (15 and 30 mg/kg, IP, one-day pre-surgery and continued for 3 weeks) on sporadic streptozotocin (STZ)-induced AD in rats. It was found that crocin at 30 mg/kg exhibited higher correct choices and lower errors in Y-maze test than control rats. Additionally, crocin significantly attenuated learning and memory impairment in passive avoidance test (61).

Moreover, crocin and crocetin, were found to provide neuroprotection by inhibiting lpopolysaccharides (LPS)-induced nitric oxide (NO) release from cultured rat brain microglial cells. These compounds reduced the LPS-induced productions of tumor necrosis factor-α (TNF-α), interleukin-1β (IL-1β), and intracellular ROS. Crocin and crocetin also effectively reduced LPS-stimulated NF-κB activation. In addition, crocin could reduce NO release from microglia stimulated by interferon-γ (IFNγ) and Aβ. In organotypic hippocampal slice cultures, both compounds inhibited the effect of LPS –induced hippocampal cell death (33).

In a 22-week, double-blind, randomized clinical trial, fifty-four 55 years of age or older patients with mild-to-moderate AD received either ethanol extract of saffron (15 mg twice per day) or donepezil (5 mg twice per day). It was found that saffron was effective similar to donepezil as evidenced by the improvements in both the Alzheimer’s disease assessment scale—cognitive subscale (ADAS-cog), and clinical dementia rating scale—sums of boxes (CDR-SB) measures in the saffron group (62).

In another randomized double-blind trial, 68 moderate to severe AD patients (Mini-Mental State Examination score of 8–14) received memantine (20 mg/day) or ethanolic extract of saffron (30 mg/day) for one year. Participants were evaluated every month by severe cognitive impairment rating scale (SCIRS) and functional assessment staging (FAST). The results showed that the effect for time × treatment interaction on SCIRS scores for saffron and memantine groups was not significant. During the 12-months period of this trial, saffron- and memantine-treated patients experienced 9.18% and 7.79% decrease in the SCIRS scores, respectively. Moreover, no significant difference between the two groups in the scores changes from baseline to the endpoint on SCIRS and FAST was observed (63).

***Cuminum cyminum*** **Linn.**

Dried fruits of *C. cyminum* commonly known as Cumin seeds are traditionally used as a spice and flavoring agent and a medicine. 

Aqueous extract of cumin fruit (50 μg/ml) has been shown to possess AChE inhibitory activity of 76.90±0.003% based on Ellman’s method (64). Koppula *et al*. reported that oral administration of aqueous extract of cumin (at 100, 200, and 300 mg/kg/day) significantly attenuated scopolamine-induced amnesia in rats using conditioned avoidance response (CAR) model by Cook’s pole climbing apparatus. This memory-enhancing activity was determined by improved acquisition, retention, and recovery in extract-treated rats compared to control group. The extract also significantly inhibited lipid peroxidation in both rat liver and brain in comparison with ascorbic acid, a known antioxidant (65). 

Oral administration of aqueous extract of cumin (100, 200 and 300 mg/kg) for three weeks significantly improved locomotor and cognitive deficits induced by 1-methyl-4 phenyl-1, 2, 3, 6-tetrahydropyridine (MPTP) in mice. Cumin also significantly improved MPTP-induced decrease in antioxidant enzyme levels (superoxide dismutase (SOD) and catalase) and inhibited lipid peroxidation in mice brain tissues (66). Cumin essential oil and its n-hexane fraction strongly inhibited α-SN aggregation in a concentration-dependent manner. Cuminaldehyde (**9**), the main component of the cumin oil and its n-hexane fraction, was shown to inhibit the fibrillation process possibly by preventing the elongation stage of the fibrillation. The carboxaldehyde structure of this compound plays important role in its potent activity. Cuminaldehyde has no toxic effects on PC12 cells (67).

***Ficus carica*** **L.**

Fig is the edible fruit of *F. carica* from Moraceae. This well-known Mediterranean fruit has long been used in fresh or dried forms and to prepare preserves and foods. 

Essa *et al*. investigated the effects of a 15 months dietary supplementation of APPsw/Tg2576 mice (Tg mice; which show age-related memory and learning impairment as well as Aβ accumulation and serve as a mice model of AD) with pomegranate, figs, or dates on suppressing inflammatory cytokines in brain. It was found that the diet supplements significantly decreased the enhanced levels of inflammatory cytokines (IL-1β, IL-2, IL-3, IL-4, IL-5, IL-6, IL-9, IL-10, TNF-α and eotaxin). In addition, remarkable delays in the formation of senile plaques, as determined by decreasing the levels of brain Aβ1–40 and Aβ1–42 was observed (68). In another study, the effect of dietary supplementation of Tg mice with 4% figs on the memory, anxiety, and learning skills using the Morris water maze test, rota-rod test, elevated plus maze test, and open-field test was studied. Tg mice fed a control diet exhibited significant memory deficits, increased anxiety-related behavior, and severe impairment in spatial, position discrimination learning ability, and motor coordination compared to the Tg mice fed 4% fig diet (69). Four% figs fed Tg mice also significantly showed attenuated oxidative damage, as indicated by decreased levels of lipid peroxidation and protein carbonyls and restoration of antioxidant status. Moreover, deranged activities of membrane bound enzymes (Na^+^ K^+^ ATPase and AChE) in Tg mice brain regions was restored by figs treatment (70). In addition, γ-sitosterol (**10**), a phytosterol found abundantly in fig mesocarp, demonstrated potent ROS scavenging activities (71). 

***Fish***

Lim *et al*. evaluated the effects of dietary docosahexaenoic acid (DHA (**11**); an omega-3 polyunsaturated fatty acid (PUFA) abundant in fish) on APP processing and amyloid formation in APPsw (Tg2576) transgenic mice. It was shown that high-DHA diet could significantly reduce total Aβ by more than 70% when compared with low-DHA or control diets. Moreover, DHA decreased Aβ42 levels compared to the control chow diet. Image analysis of brain sections revealed a 40.3% decrease in overall plaque burden (40-50% reductions in the hippocampus and parietal cortex). DHA also regulated APP changes by reducing both α- and β-APP C-terminal fragment products and full-length APP (72). In a clinical study, it was found that consumption of fish once per week or more lowered the risk of AD by 60%. Total intake of omega-3 polyunsaturated fatty acids (e.g. DHA) was associated with reduced risk of AD (73). It is evidenced that DHA levels are lower in serum and brains of AD patients, which can be the result of low dietary intake and/or oxidation of PUFA (72). 

The results of another study revealed that intake of fatty fish, which is a rich source of DHA, more than twice per week was associated with a reduced risk of dementia by 28% (95% CI: 0.51 to 1.02), and AD by 41% (95% CI: 0.36 to 0.95) compared to fish consumption less than once per month especially in people without the APOE epsilon four allele (74). In the Zutphen Elderly Study, fish consumption was associated with less 5-year subsequent cognitive impairment. Moreover, a linear fashion was observed for the relation between the consumption of (ecosapentaenoic acid) EPA+DHA and cognitive decline. The intake of approximately 380 mg/d of EPA+DHA was associated with a 1.1-point less cognitive decline in elderly men (75). 

***Foeniculum vulgare*** **L.**

Fennel seeds (Apiaceae) are commonly used as a spice and flavoring agent in Persian cuisine. It is also used to alleviate a wide spectrum of illnesses especially CNS problems. 

Administration of fennel extract (50, 100 and 200 mg/kg/day) significantly reduced the stress-induced urinary levels of vanillylmandelic acid in rats. Moreover, fennel dose-dependently improved scopolamine-induced memory deficits in rats. The extract also potently inhibited lipid peroxidation in both rat liver and brain homogenates to a greater extent than the standard antioxidant, ascorbic acid (76).

Administration of methanolic extract of the whole fennel plant (200 mg/kg, p.o.) for 8 days ameliorated scopolamine-induced amnesia and aging-induced memory deficits in mice compared to the control group. In the passive avoidance test, fennel extract could increase step-down latency (SDL). Moreover, the extract (50, 100 and 200 mg/kg, PO) significantly increased acetylcholinesterase inhibition in mice (77). Bhatti *et al*. investigated the neuroprotective effects of ethanol extract of fennel seeds in a lead-induced neurotoxicity mice model. The results showed that the defected expression levels of oxidative stress markers (SOD1 and peroxiredoxin-6) and the three isoforms of APP (APP common, 770 and 695), in the cortex and hippocampus were significantly normalized in mice treated by fennel extract (specifically at dose of 200 mg/kg/day). Moreover, the morphological impairments of cortical neurons were significantly ameliorated by fennel extracts (78). Anethole (**12**), major component of fennel essential oil has been found to possess AChE and BChE inhibitory activity with IC_50_ values of 39.89 ± 0.32 μg/ml and 75.35 ± 1.47 μg/ml (79).

***Honey***

Akanmu *et al*. evaluated the neuropharmacological effects of three types of Nigerian honeys (10%, 20% and 40% v/v, PO). The results showed that honey significantly decreased locomotion and rearing behaviors in novelty-induced behaviors (NIB) and amphetamine-induced locomotor activity tests compared to the control animals which suggests central inhibitory effects (80). 

Abdulmajeed *et al*. investigated the neuroprotective effects of honey against lead-induced neurotoxicity. The results showed that co-administration of honey with lead inhibited neurotoxicity and improved memory function as evidenced by shortened latency period and elongated time spent in target quadrant in honey-fed rats compared to the lead-exposed animals using Morris water maze. In open field test, honey could increase locomotion, exploration and decrease anxiety in lead-exposed animals. Honey increased brain SOD, glutathione S-transferases (GST) and glutathione (GSH) activities but had no significant effect on MDA level. Therefore, neuroprotective effects of honey might be associated with its ability to enhance antioxidant capacity (81).

In another experiment, the effects of Malaysian Tualang honey on hippocampal morphology and memory performance in stressed ovariectomized rats were studied. Tualang honey could improve short-term and long-term memory and enhance the neuronal proliferation in several regions of hippocampus (CA2, CA3 and DG) (82). Honey also restored normal structure of hippocampal cells and prevented loss of neurons in rats with chronic cerebral hypoperfusion (83). In another animal cognition experiment (Y maze), honey-fed rats could better recognize the novel arm at 9 and 12 months compared to sugar-free or sucrose-fed animals. This suggest that honey can delay or prevent aging-related memory loss (84).

***Juglans regia*** **L.**

Walnut is the edible seed of *J. regia* from Juglandaceae. Walnut is widely used in Persian cuisine. It is also recommended to prevent and treat memory loss in TPM. 

Aqueous tea infusion from flowers and leaves of walnut have been shown to exhibit moderate AChE Inhibitory activity of 45% at 1.36 g/l. Moreover, high antioxidant activity was observed for walnut using DPPH radical scavenging, LDL oxidation, beta-carotene bleaching and Rancimat tests (85).

Neha *et al*. investigated the inhibitory effect of walnut extract on Aß fibrillization using Thioflavin T fluorescence spectroscopy and electron microscopy. The walnut methanolic extract inhibited Aß fibrillization in a concentration and time- dependent manner (over 90% inhibition with 5 ml of extract). Interestingly, the extract could defibrillize the preformed fibrils of Aβ (91.6% with10 ml of extract). These results suggest that walnuts may reduce the risk or delay the onset of AD by maintaining the solubility of Aß (20).

**Table 1:** Plant-Based Foods Recommended by TPM for treatment of dementia and memory impairment

**Scientific Names**	**TPM Names**	**Part used**	**References**
*Allium sativum* L.	Seer	Bulb	(27)
*Alpinia officinarum* Hance	Khowlanjan	Rhizome	(26)
*Brassica nigra* L.	Khardal	Seeds	(28)
*Cicer arietum *L.	Nokhod, Homs	Seeds decoction	(10, 26)
*Cinnamomum cassia* (L.) J.Presl	Salikheh	Root bark	(9, 26)
*Cocos nucifera* L.	Nargeel	Seeds	(26)
*Corylus avellana* L.	Fandoq	Seeds	(8, 26)
*Crocus sativus *L.	Zaafaran	Stigma	(9, 10)
*Cuminum cyminum *Linn.	Cammun	Seeds	(10)
*Ficus carica *L.	Anjeer	Fruits (mature syconium)	(26)
*Foeniculum vulgare *L.	Razianeh	Seeds	(9)
*Hordeum vulgare *L.	Jow	Seeds	(10)
*Juglans regia *L.	Gerdu, Jowz	Seeds	(25, 26)
*Melissa officinalis *L.	Badranjbuyeh	Seeds, Leaves	(26)
*Myristica fragrance* Haut.	Jowz buya	Seeds	(26)
Persian wheat noodles	Reshte	-	(27)
*Pinus gerardiana* Wall. ex D.Don	Chalghuzeh	Nuts	(8, 26)
*Piper nigrum *L.	Felfel syah	Fruits	(8-10, 25, 28)
*Pistacia* *atlantica* Desf.	Baneh	Seeds	(26)
*Pistacia vera *L.	Pesteh	Seeds	(8, 26)
*Prunus amygdalus Batsch*	Badam	Seeds	(8, 26)
*Rosa damascna* Mill.	Gol-e-sorkh	Hydrolate	(26, 29)
*Syzygium aromaticum* (L.) Merr. & L.M.Perry	Qaranfol	Buds	(9)
*Trachyspermum ammi* (L.) Sprague	Zenyan, Naankhah	Seeds	(26)
*Trigonella foenum-graecum* L.	Shanbalileh, holbeh	Seeds	(26)
*Vitis vinifera* L.	Maviz	Red raisin, vinegar	(9, 26, 27)
*Zingiber officinale* Roscoe	Zanjabeel	Rhizome	(8, 9, 25)

***Melissa officinalis*** **L.**

Lemon balm, a well-known vegetable and medicinal plant from Lamiaceae, has been traditionally used to cure dementia and amnesia (86). Ethanol extract of lemon balm exhibited AChE inhibitory activity (87) with a potency of 1.72±0.16 μg equivalents of physostigmine/mg of the extract. AChE inhibitory guided fractionation of the same extract revealed that most fractions have more potent inhibitory activities than the crude extract. The most potent fraction contains mostly cis- and trans-rosmarinic acid (**13**) isomers and a rosmarinic acid derivative with a methyl ester or a methoxy group (88). Moreover, ethyl acetate fraction of the hydroalcoholic extract of lemon balm contains high amounts of flavonoid and possessed antioxidant and AChE inhibitory properties (89). Lemon balm essential oil also showed AChE inhibition in a dose-dependent manner (87). 

Stimulation of acetylcholine receptors has been shown to be another strategy for the treatment of AD (90). An 80% ethanol extract of lemon balm could displace [3H]-(N)-nicotine and [3H]-(N)-scopolamine (the ligand for muscarinic receptors) from brain cell membranes containing nicotinic or muscarinic ACh receptors (91).

Ethanol extract of lemon balm improved learning and memory of naive rats and attenuated scopolamine-induced learning impairment similar to the other cholinesterase inhibitors. AChE inhibition has also occurred in both naive and scopolamine-induced rats (92). Luteolin (**14**), a major component of lemon balm could improve scopolamine-induced memory impairment in passive avoidance task in rats. It also attenuated Aβ -induced memory deficiency observed in water maze task (93, 94). Ursolic acid (**15**), another important component of the plant improved age-related cognitive decline by activating the antioxidant enzymes and suppressing lipid peroxidation (95). 

In a clinical trial, the effects of lemon balm on cognitive performance and mood of healthy volunteers were investigated. It was shown that acute administration of lemon balm ethanol extract (600 mg) modulated mood and cognitive performance (96). It was suggested that cholinergic receptor-binding activities of lemon balm in the human cerebral cortex might be responsible for the observed effects (97). Similarly, a 16-week placebo-controlled clinical trial on 42 patients with mild-to-moderate AD showed that hydroalcoholic extract of lemon balm (60 drops/day) containing 500 μg citral/ml improved agitation and cognitive and behavioral functions (98).

***Milk***

Whey proteins consist of α-lactoalbumin, β-lactoglobulin, immunoglobulin, albumin, *etc.* from cow’s milk. Whey proteins have been found to elevate the levels of glutathione in serum (99). Glutathione has protecting effects against ROS, redox metal ions, and reactive lipid peroxidation products and other molecules involved in AD pathology (100). Albumin, α-lactalbumin, and β-lactoglobulin are also reported to suppress the fibrillation of Aβ(1–40) peptide by non-specifically binding to Aβ, stabilizing its random coils, and reducing its cytotoxicity (101).

***Myristica fragrans*** **Haut.**

*M. fragrans* is an evergreen tree from Myristicaceae which bears fragrant seeds commonly known as nutmeg. Nutmeg is a spice which is widely used in Asian cuisine and confectionary. It is also a beneficial medicinal plant used in TPM.

Hydroalcohol extract of nutmeg could inhibit 50% of AChE activity at concentrations of 133.28±11.26 µg/ml. Oral administration of n-hexane extract of nutmeg seeds (5 mg/kg p.o. for 3 days), significantly decreased AChE activity in mice as compared with control groups (102). Oral treatment of young and aged mice with n-hexane nutmeg extract at the dose of 5 mg/kg for 3 days significantly improved learning and memory. The extract also reversed scopolamine- and diazepam-induced learning and memory impairment of young mice and improved learning and retention capacities of both young and aged mice (103). Van der Auwera *et al*. investigated the effect of a ketogenic diet (KD; high saturated fat/low carbohydrate) rich in myristic acid (**16**), a saturated fatty acid found in nutmeg, on a transgenic mouse model of AD. Mice fed the KD exhibited remarkable increase in serum ketone body levels, as measured by β-hydroxybutyrate (3.85 ± 2.6 mM), compared to standard diet fed animals (0.29 ± 0.06 mM). Moreover, the KD, significantly reduced total brain Aβ levels by approximately 25%. However, the KD diet did not alter behavioral impairment in AD mice (104).

***Piper nigrum*** **L.**

*Piper nigrum *(black pepper) is a well-known spice from Piperaceae and is used to add a pleasant pungent flavor to various dishes. Piperine (**17**) (1-piperoylpiperidine), an alkaloid, is the principle bioactive component of black pepper and is responsible for its pungency (55). 

Ingkaninan *et al*. reported that at the concentration of 0.1 mg/ml, black pepper methanolic extract showed 58.02±3.83 inhibitory activity on AChE using Ellman’s colorimetric method (105). In another study, dichloromethane extract of black pepper peeled seeds demonstrated approximately 23% AChE inhibition at the concentration of 100 μg/ml (106).

Chonpathompikunlert *et al*. demonstrated that piperine could significantly improve ethylcholine aziridinium ion (AF64A)-induced memory impairment and neurodegeneration in rat’s hippocampus after oral administration of 5, 10 and 20 mg/kg. These effects have been shown to be related to a decrease in lipid peroxidation and AChE enzyme and an increase in neuron density (107).

***Pistacia vera*** **L.**

*Pistacia vera* seeds, commonly known as pistachio, are widely used as edible nuts and as food and confectionary additive. Pistachio also has recommended to enhance memory and cognition ability in TPM. In an experiment, pistachio supplementation improved memory and motor abilities in cisplatin- or vincristine-induced neurotoxicity in rats. However, pistachio could not reverse cisplatin and vincristine induced increase in the latency of response to nociception (108). In another study, pistachio extract (10, 50, and 100 mg/kg/day for 14 days) administration in rats showed a significant increase in the latency to enter the dark room compared to control group. Moreover, pistachio decreased the time spent in the dark room indicating the beneficial effects of pistachio to improve learning and memory (109). Pistachio is a rich source of γ-tocopherol and other antioxidants. Moreover, the skin of nuts contains high amounts of resveratrol (**18**) (110). These compounds have been proved to be effective in attenuating AD symptoms (see raisin section). 

***Prunus amygdalus*** **Batsch**

In an experiment, oral administration of the paste of almond nuts (150, 300 and 600 mg/kg) for 7 and 14 consecutive days to rats significantly improved scopolamine-induced amnesia, as determined by a decrease in the transfer latency in the EPM task and step-down latency in the passive avoidance task. Almond also reduced the ChE activity in rats brain (111). Moreover, Batool *et al*., demonstrated that almond supplementation (400 mg/kg/day) for 28 days attenuated cadmium-induced memory loss in rats (112). 

α-Tocopherol (vitamin E) is one the important compounds present in almond (113). It has been reported that the CSF and serum α-tocopherol levels are lower in AD patients (114). In a double-blind, placebo-controlled, randomized, multicenter trial in AD patients with moderate severity, administration of vitamin E (2000 IU /day for two years) could significantly slow the progression of the disease (115). 

***Red raisin and vinegar*** **(** ***Vitis vinifera*** **L.)**

Gol *et al*. investigated the effects of raisin consumption (6 g of raisin per rat for 60 days) on aluminium chloride (AlCl_3_)-induced AD in rats using Morris water task and passive avoidance test. Both AD and control rats were treated with raisin. The results showed that AlCl_3_ exposure significantly decreased the memory in both tests, but spatial memory was significantly improved in rats treated with raisin + AlCl_3_. Also, AlCl_3_ significantly enhanced MDA and decreased ferric reducing ability of plasma [ferric reducing/antioxidant power (FRAP)], while raisin decreased MDA and increased FRAP (116). Moreover, it has been shown that polyphenols isolated from grape seeds reduced Aβ formation, inhibited Aβ aggregation, and protected against Aβ neurotoxicity *in vitro* (117).

Resveratrol (trans-3,4′,5-trihydroxystilbene), a polyphenol mainly found in grapes, markedly lowers the levels of secreted and intracellular Aβ peptides produced from different cell lines. Resveratrol enhances intracellular degradation of Aβ via mechanisms involving the proteasome and has no effect on Aβ production suggesting a proteasome-dependent anti-amyloidogenic activity for this compound (118).

In Tg mice, resveratrol treatment (a diet containing 0.2% resveratrol for 45 days) reduced the amyloid plaque pathology in cortex, striatum and hypothalamus, decreased brain GSH and increased its precursor product cysteine (119). It has been reported that long-term resveratrol supplements (1 g/kg of diet for 9 months) increased mean life expectancy and maximal life span in the age-accelerated mice (SAMP8) and in their control, SAMR1 mice. In addition, dietary resveratrol activates AMP-activated protein kinase (AMPK) pathways and pro-survival routes such as Sirtuin 1 (SIRT1) *in vivo*. It also improved cognitive impairment, decreased the amyloid burden and reduced tau hyperphosphorylation (120). 

The effect of long-term oral resveratrol administration on Aβ protein precursor/presenilin 1 (AβPP/PS1) mice (a mouse model of AD), was investigated by Porquet *et al*. Resveratrol treatment significantly prevented memory impairment as indicated by the object recognition test. Moreover, resveratrol reduced the amyloid burden and increased mitochondrial complex IV protein levels in mouse brain mainly mediated by enhancing the activation of SIRT1 and AMPK pathways (121).

***Rosa damascna*** **Mill.**

The rose oil exhibited inhibitory effects against AChE (60.86 ± 1.99%) and BChE (51.08 ± 1.70%) at 1 mg/ml. Phenylethyl alcohol, a minor component of rose oil, potently inhibited AChE (73.87%) and BChE (91.50%). Major components of rose oil e.g. geraniol (**19**), nerol (**20**), (−)-β-citronellol (**21)** and (+)-β-citronellol (**22**) also exhibited moderate to potent BChE inhibition at 0.01-1 mg/ml (122). 

In a rat model of Aβ-induced AD, a methanolic extract of rose flowers significantly improved the spatial and long-term memories in a dose-dependent manner (123). Mohammadpour *et al*. investigated the effect of hydroalcoholic extract of rose flowers (50 and 250 mg/kg for two weeks) on memory performance in a scopolamine-induced memory impairment model. Rose extract treated groups in both doses showed significantly shorter traveled distance and time latency compared with scopolamine group. Time spent in target quadrant was longer at the dose of 250 mg/kg extract compared to that of scopolamine group. Moreover, both doses of the extract were able to decrease the MDA concentration and increase the thiol levels in hippocampal and cortical tissues (124). In addition, hydroalcoholic extracts of rose petals (oral gavage of 1 g/kg daily for 1 month) improved high-fat diet-induced memory deficits in male rats compared to control group as measured by the passive avoidance learning (PAL) test (125).

In a rat model of Aβ-induced AD, Esfandiary *et al*. showed that rose extract (oral doses of 300, 600, and 1,200 mg/kg/day for one month) could increase adult hippocmapal neurogensis. Adult rats receiving 600 and 1,200 mg/kg/day of rose extract generated 67% and 77% more neurons per dentate gyrus than control rats. These effects might be associated to an increase in expression of neurotrophic factors (brain-derived neurotrophic factor (BDNF), nerve growth factor (NGF), and cyclic AMP response element binding protein (CREB) transcription factor), cell proliferation and survival, and differentiation of neural stem cells to neurons. Rose extract dose-dependently increased total volume of hippocampus and DG and CA1 regions of the hippocampus which were decreased by Aβ injection. Moreover, the extract at 600 and 1,200 mg/kg/day enhanced synaptic plasticity in a dose-dependent manner (126).

In a *Caenorhabditis elegans* AD model, rose essential oil significantly improved AD-like symptoms of exogenous 5-HT-induced worm paralysis and hypersensivity in a dose-dependent manner. Rose oil alleviated the Aβ toxicity by significantly suppressing Aβ deposits and reducing the Aβ oligomers. Rose oil markedly activated the expression of GST-4 gene, which supports the involvement of SKN-1 (The *C. elegans* inducible transcription factor, a homolog of mammalian Nrf proteins, which responds to oxidative stress) signaling pathway in the observed effects of rose oil on *C. elegans *AD (127).

***Syzygium aromaticum*** **(L.) Merr. & L.M.Perry**

*S. aromaticum* (Myrtaceae) aromatic flower bud commonly known as clove is a popular spice and medicinal plant. Shekhar *et al*. investigated the effect of ethanolic extract of cloves on SIRT1 and oxidative balance in Aβ-induced toxicity. Clove extract could scavenge ROS and elevate the antioxidant enzymes (SOD, CAT and GSH). Moreover, clove treatment (2 μg/ml and 10 μg/ml) elevated the level of SIRT1 in serum of AD patients and down-regulated γ-secretase in Aβ_25-35_induced neurotoxic cells (128).

Pant *et al*. evaluated the effect of clove oil on oxidative stress, lifespan, mobility, and the expression of aging-related proteins using *C. elegans* model. The clove oil (10 ppm) could significantly extend mean lifespan in worms by 21.4% under normal condition and by 63% under juglone-induced oxidative stress condition. The extension of mean lifespan in mev-1 mutant (30.94 %) and up-regulation of stress response genes, gst-4 (19.38%) and sod-3 (29.7%) was also observed by clove oil treatment which is indicative of stress modulatory activity of clove oil. Moreover, clove oil reduced intracellular ROS by 26% and decreased Aβ1–42 proteotoxicity (129). 

***Trachyspermum ammi*** **(L.) Sprague**

*Trachyspermum ammi* (Ajowan) is an annual plant of Apiaceae. It is used as a common spice and a medicinal plant in Persian culture.

Soni *et al*. investigated the effects of supplementation of ajowan seed’s powder (at the dose of 0.5%, 1.0% and 2.0% w/w of normal diet) for 10 successive days on learning and memory of mice using passive avoidance paradigm and object recognition task (ORT). Moreover, the brain AChE activity, brain MDA, GSH and nitrite were evaluated. Alprazolam, scopolamine and electroshock were used to induce amnesia. Ajowan treatment significantly increased step down latency of passive avoidance response and enhanced discrimination index of ORT in animals with or without amnesia when compared to control groups. In addition, ajowan administration significantly decreased brain AChE activity and MDA and nitrite levels while increasing brain GSH level suggesting a decrease in oxidative damage (130). 

***Trigonella foenum-graecum*** **L.**

Satheeshkumar *et al*. showed that the ethyl acetate fraction of fenugreek seed extract and its total alkaloid fraction possess moderate AChE inhibitory activity with IC_50_ values of 53.00±17.33 μg/ml and 9.23±6.08 μg/ml, respectively. As standard, galanthamine showed AChE inhibition with an IC_50_ value of 1.27±0.21 μM (131).

Prema *et al*. demonstrated that chronic administration of AlCl_3_ to rats induced significant learning and memory deficit, oxidative stress, and alterations in levels of insulin-degrading enzyme (IDE) and cyclin-dependent kinase 5 (CDK5) (enzymes involved in the metabolism of tau and amyloid), pTau, glial fibrillary acidic protein (GFAP), ionized calcium-binding adapter molecule 1 (IBA1), IL-1β, IL-6, TNF-α, iNOS, NF-κB, COX-2, brain-derived neurotrophic factor (BDNF), and STAT3. Co-administration of fenugreek seed powder significantly attenuated the AlCl_3_ induced memory and learning impairment, amyloid and tau pathology, oxidative stress, and inflammatory responses (132).

***Zingiber officinale*** **Roscoe**

Oboh *et al*. investigated AChE inhibitory activities of water extracts of red ginger (*Z. officinale* var. *Rubra*) and white ginger (*Z. officinale* var. *Roscoe*), *in vitro*. Both extracts had AChE inhibitory activities in a dose-dependent manner at the concentrations of 0–6.76 mg/ml. The IC_50_ values for white and red gingers were 2.86±0.07 and 3.03±0.04 (mg/ml), respectively. Both extracts significantly decreased sodium nitroprusside (SNP) and quinolinic acid (QA)-induced elevated MDA contents in rat brain in a dose-dependent manner which suggests their inhibitory effects on lipid peroxidation (133).

In an *in vivo* study, rats were treated orally with ethanolic extract of ginger rhizome 14 days before and 21 days after the permanent occlusion of right middle cerebral artery (MCAO). The results showed that cognitive function were significantly improved as evidenced by a decrease in escape latency throughout the experimental period using the Morris water maze test. Neuronal density in hippocampus of rats receiving ginger extract was increased only in CA3 at dose of 100 mg/kg, whereas it was increased in both dentate gyrus and CA3 at 200 mg/kg. Moreover, the brain infarct volume in ginger group was significantly decreased. Ginger extract could also decrease oxidative stress and lipid peroxidation level as determined by increasing the activity of SOD, CAT and glutathione peroxidase in cerebral cortex and hippocampus (134). Ginger methanolic extract was able to significantly increase the cell viability against Aβ-induced toxicity in primary adult rat hippocampal cell culture using MTT assay. Ginger could also inhibit the formation of Aβ oligomers and defibrillize the preformed oligomers in thioflavin T binding studies (135).

Ghayur *et al*. reported that a 70% aqueous/methanolic extract of ginger showed a spasmogenic effect (0.03–5.00 mg/ml) on isolated rat stomach fundus. The spasmogenic effect was insensitive to inhibition by hexamethonium (a ganglion blocker) and methysergide (a non-selective serotonin antagonist), but sensitive to atropine (a muscarinic receptor inhibitor), indicating stimulation of muscarinic receptors. Moreover, ginger was shown to possessed Ca^2+ ^antagonist and specific BuChE inhibitory activities. Ginger major compounds 6-gingerol and 6-shogaol exhibited spasmolytic activities possibly through Ca^2+ ^antagonism. 6-gingerol also showed BuChE-specific inhibitory properties *in vitro*. (136). Gingerol (**23**) at doses of 10 and 20 mg/kg was able to improve the cognitive and behavioral impairment and AD-like pathology in a mice model of STZ-induced sporadic AD. These effects were accompanied by an increase in α-secretase activity and a decrease in cerebral Aβ-42, β- secretase, APH1a activity and COX-2-linked neuro-inflammation (22). 

In a randomized, placebo-controlled clinical trial, the effect of ethanol extract of ginger, on the cognitive function of middle-aged, healthy women was studied using computerized battery tests and the auditory event-related potentials in N100 and P300 amplitudes and latencies. Sixty participants received a placebo or standardized extract at doses of 400 and 800 mg once daily for 2 months. The results demonstrated that the ginger-treated groups had significantly decreased P300 latencies, enhanced N100 and P300 amplitudes, and exhibited improved working memory (137). The results of the mentioned studies suggest that the cognitive enhancing effects of ginger might be partly via alteration of both the cholinergic and the monoamine systems in various brain areas, including the prefrontal cortex and hippocampus.

## Discussion

Multiple lines of evidence, point out the importance of diet in prevention and improvement of AD (9). According to epidemiological studies, adherence to especial diets can be associated to reduced risk of cognitive impairment, vascular dementia and AD (138). Traditional Persian Medicine as one of the widest practiced medical systems, not only provide with experienced therapeutic strategies for the treatment of AD, but also offers dietary recommendations for prevention and slowing down the progression of the disease. Most of the TPM-recommended dietary items have been shown to improve memory and cognitive decline and possess anti-amyloidogenic, *etc.* activities. Among them, garlic and its compounds, S-allyl-cysteine and diallyl-disulfide, coconut oil, saffron and its major compounds, crocin and crocetin, honey, fish, lemon balm and its major compound rosmarinic acid, raisin and resveratrol, rose flowers and geraniol, ginger and its 6-gingerol and 6-shogaol, cumin and its main component cuminaldehyde have been found to possess stronger anti-AD activities. Most of these food items and compounds exhibited remarkable antioxidant and AChE inhibitory activities and decreased oxidative stress and lipid peroxidation level (134). In animal AD models, they showed anti-amyloidogenic effects, reduced cerebral plaques and soluble and fibrillar Aβ-species, suppressed cerebral inflammation and alterations in tau protein and inhibited Aβ-induced apoptosis through various mechanisms including elevating sAPPa levels, decreasing Bax/Bcl-2 ratio and cleaved caspase-3 level, decreasing in Aβ40 and Aβ42, reducing NO release from microglia, activation of SIRT1 and AMPK pathways, etc. (33, 37, 60). The TPM-recommended foods also increased neuronal density and total volume of hippocampus (126, 134) and improved the cognitive and behavioral impairment and AD-like pathology in animal experiments (35). Notably, anti-AD effects of a number of TPM-recommended food items including ginger, saffron, fish, coconut oil and lemon balm have been supported by epidemiological studies. 

Mediterranean diet as one of the most distinguished healthy diets is characterized by high intake of vegetables, fruits, legumes, cereals, nuts, fish and olive oil (containing PUFA), but low intake of saturated fatty acids, dairy products, meat and poultry (139). There is a large volume of evidence supporting an association between the adherence to MD and slower rates of cognitive decline, improvements in cognitive performance and reduced incidence of AD (139, 140). Interestingly, there are close similarities between TPM anti-AD diet and the typical MD. For instance, vegetables like garlic, lemon balm and fenugreek; honey, fish, spices like saffron, cumin, mustard and fennel; legumes especially chickpea; nuts especially walnuts, almond and pistachio are highly consumed in both diets. 

The use of egg, fish eggs, gout and cow milk, bird’s meat and brain and especial food items including *Pistacia atlantica*, *Pistacia gerardiana*, ‘reshteh’ (Persian whole wheat noodles) and barley soup has been recommended by TPM as dietary items to prevent AD or to be included in AD patient’s diet (11, 12, 15). Pharmacological and clinical evidence supporting anti-AD effects of these items and a number of other TPM-foods are rare. Therefore, future animal and human studies evaluating their potential effects in prevention and alleviation of AD are needed. 

Interestingly, excessive consumption of some vegetables and foodstuffs such as onion (*Allium cepa*), coriander (*Coriandrum sativum*), lettuce (*Lactuca sativa*), broad bean (*Vicia faba*), (15) have been ruled out from anti-AD TPM diet. It is necessary to mention that a number of the most potent anti-Alzheimer food items including garlic, walnut and mustard have been considered harmful by TPM in some other CNS disorders such as epilepsy and vertigo due to their effects on the raise of detrimental, pro-epileptic humors into the brain. (27). However, no pharmacological data supporting this effect exist which necessitates conducting additional studies devoted to epileptonic properties of these plants. Moreover, a number of activities including excessive sexual activity, exercise or heavy physical activity after meals, drinking iced water, *etc*. are considered to trigger AD. Therefore, considering these recommendations in AD patients’ lifestyle and studying their impact would be of value to expand our current knowledge on etiology and treatment of AD.

## Conclusion

Given the important role of different traditional medicine systems in discovering new therapeutic strategies, medicines and nutraceuticals for curing ailments, considering TPM anti-AD dietary recommendations in future research would be helpful. Moreover, further research on molecular and cellular mechanisms of actions, clinical effects, adverse reactions, optimum doses and pharmacokinetic profile of the discussed food items and their bioactive compounds, can pave the way to more effective new anti-AD product development.

## Conflicts of Interest

The authors report no conflict of interest.
